# Would the Physicians Eventually Obsolete the Liver Biopsy for the Assessment of Liver Fibrosis?

**DOI:** 10.5812/hepatmon.6227

**Published:** 2012-05-30

**Authors:** Hadi Parsian, Maryam Alizadeh, Hajar Negahdar

**Affiliations:** 1 Cellular and Molecular Biology Research Center, Babol University of Medical Sciences, Babol, IR Iran; 2 Departments of Biochemistry and Biophysics, Babol University of Medical Sciences, Babol, IR Iran

**Keywords:** Liver Biopsy, Physicians, Fibrosis

Dear Editor,

In recent article which is published in your journal Crisan D et al. [1] has been reported an interesting article about noninvasive assessment of liver fibrosis. They evaluated six serum marker panels (APRI, Forns, Fib-4, Hepascore, Fibro Test, Fibro meter) and transient elastography (TE) alone or in combination, for prediction of liver fibrosis stages in 446 chronic hepatitis C (CHC) patients. In addition they evaluated whether the combination of serum panels with TE could increase the diagnostic accuracy of liver fibrosis assessment or not. The authors concluded that combination of some of previously mentioned serum marker panels with TE, increases the diagnostic accuracy of non-invasive methods for the assessment of liver fibrosis stage. Studies such as previously mentioned article are very important, because the findings would help us to improve our knowledge about the staging of liver fibrosis. Nowadays the list of serum marker algorithms for assessment of liver fibrosis is increasing. According to the literature, there are various algorithms for noninvasive assessment of liver fibrosis in patients with viral chronic hepatitis C and the most well-known of them are: Fibro test, Forns, APRI, FIB-4, Hepascore, Fibro meter, ELF, Fibro scan [2][3][4][5][6][7][8]. Certainly in the future, new serum marker panels will add to this list. In addition some techniques such as transient elastography will help physicians in the exact estimation of the liver fibrosis stage [9]. In Most of the published studies these serum markers panels were used in order to assess liver fibrosis in patients with chronic hepatitis, particularly chronic hepatitis C. simultaneously It is recommended that combination of these markers done in other types of liver fibrosis diseases including chronic hepatitis B, autoimmune hepatitis, non-alcoholic fatty liver disease and the others.

Now in calculation of the severity of liver fibrosis, liver biopsy stayed the gold standard; however the acceptability of this method by patients is low. In addition this method has some major limitation and its limitations made it a non-favorable method [6]. But it seems that the major benefit of liver biopsy is its applicability for assessment of liver fibrosis stages in all liver fibrosis diseases. In addition by this method, physicians are able to determine the grade of necro inflammatory injury of liver. In clinic, we should able to diagnose liver fibrosis stage and also grade of liver necro inflammatory injuries (because of any etiology) with a simple and noninvasive method. To reach to this issue many studies have been done and various noninvasive serum marker panels were compared. Most of these studies compared the diagnostic accuracy of noninvasive markers with liver fibrosis and our knowledge about liver necro inflammatory injuries is relatively low. As a try to evaluate the diagnostic accuracy of some of these biomarkers in liver fibrosis with the etiology other than viral hepatitis C, we examined the efficacy of some of these serum marker panels in chronic hepatitis C, B and autoimmune hepatitis patients’ simultaneously. The results showed that in spite of the differences in the etiology of liver fibrosis, measurement of serum laminin (LN), N terminal peptide of procollagen type III (PIIINP), AAR (AST to ALT), AP (Age-platelet index), APRI, FIB-4 and FibroQ score (derived from this simple formula: Fibro Q = [(10 × age (years) × AST × PT INR)/ (PLT × ALT)]) concentrations can discriminate between patients with liver fibrosis and healthy individuals and it seems only the extracellular matrix components i.e. LN and PIIINP performed better at excluding advanced fibrosis than mild fibrosis. [10]. In conclusion we think that it is need for some big studies to examined the efficacy of these serum marker panels, alone or in combination, with newer noninvasive tests (but more expensive such as TE), in various etiology of liver disease. Perhaps with finding upright results in various etiologies of liver diseases, eventually we can obsolete liver biopsy as the gold standard for assessment of liver biopsy.
